# A case report of fatal feline babesiosis caused by *Babesia canis* in north western Spain

**DOI:** 10.1186/s12917-022-03287-4

**Published:** 2022-05-14

**Authors:** Susana Remesar, Jose Luis Arnal, Andrea Gómez, Alberto Prieto, David García-Dios, Alfredo Benito, Rosario Panadero, Patrocinio Morrondo, Pablo Díaz

**Affiliations:** 1 Investigación en Sanidad Animal: Galicia (Grupo INVESAGA), Facultade de Veterinaria, Universidade de Santiago de Compostela, Pabellón I. Campus Universitario S/N, 27002 Lugo, Spain; 2 Exopol SL, Saragossa, Spain; 3 Centro Veterinario Meira, Meira, Spain

**Keywords:** *Babesia canis*, Feline babesiosis, Cat, Spain, Case report

## Abstract

**Background:**

In Europe, *Babesia* infections in cats are sporadic and only partial knowledge is currently available since the number of described cases including both the clinical presentation and the molecular identification of the *Babesia* species involved is limited. In the present case report, the clinical signs, the epidemiological data and the molecular results suggest that this is the first reported fatal case of feline babesiosis caused by *Babesia canis*.

**Case presentation:**

A six month old female European shorthair cat from north-western Spain died after being hospitalized for two days. This animal was pregnant and showed anorexia, lethargy, weakness, jaundice and fever with increased respiratory and heart rates. Haematological analysis revealed haemolytic regenerative anaemia, thrombocytopenia and leukocytosis. The presence of piroplasms was assessed using a PCR targeting the 18S rRNA gene of *Babesia* spp. and *Theileria* spp.; the sample resulted positive and *B. canis* was identified by DNA sequence analysis. The possible existence of co-infections with other vector-borne pathogens such as *Anaplasma* spp., *Bartonella* spp., *Borrelia burgdorferi* s.l., *Cytauxzoon* spp., *Ehrlichia* spp., *Hepatozoon canis*, *Mycoplasma* spp. or *Rickettsia* spp. was excluded by qPCR.

**Conclusions:**

Our results together with previous reports on *Babesia* infections in cats from Europe suggest that feline babesiosis should be included in the differential diagnosis of animals with anaemia, thrombocytopenia, anorexia and lethargy, especially in young or immunocompromised animals from endemic areas for canine babesiosis.

## Background

Feline babesiosis is a tick-borne disease caused by haemoparasites belonging to the genus *Babesia* [1]. Although more than ten *Babesia* species and subspecies have been molecularly identified in domestic cats, only a few of them have been associated with clinical disease [1]. *Babesia felis* is the most common species causing babesiosis in cats, being considered endemic in South Africa [2, 3]. Cats infected with *B. felis* usually tolerate a high parasitemia before showing any clinical sign [4], with anorexia, lethargy and weakness being the most common, and fever, splenomegaly, jaundice, emesis and respiratory signs being occasional [2]. Cats with complicated babesiosis can develop renal failure, pulmonary oedema and hepatic and neurological alterations [5].

*Babesia* infections in cats are sporadic in non-African countries. In Europe, *Babesia* positive cats have been only reported in France [6], Germany [7], Poland [8], Portugal [9–11], Spain [9] and Italy [12–15]. Nevertheless, current knowledge on feline babesiosis in European countries is only partial since the number of described cases including both the clinical presentation and the molecular identification of the *Babesia* species involved is limited. Thus, most of these positive animals were detected in epidemiological studies and no detailed data about clinical signs are available. A comprehensive analysis of these investigations revealed that most positive animals were asymptomatic or showed a mild clinical course [16] mainly characterised by fever, weakness, anorexia and anaemia [7–10, 17, 18]. Those investigations including molecular data demonstrated the presence of three *Babesia* species, namely *Babesia canis*, *Babesia vogeli* and *Babesia vulpes,* which are the main agents of canine babesiosis in this continent [10–15].

In the present case report clinical signs, epidemiological data and molecular results are provided, suggesting that this is the first report of a fatal case of feline babesiosis caused by *B. canis*.

### Case presentation

The CARE guidelines (https://www.care-statement.org/checklist) have been followed in the reporting of this case. On 10th of March 2021, a six month old female European shorthair cat arrived to a veterinary clinic located in Meira (north-western Spain) presenting anorexia, lethargy, weakness and respiratory distress; the animal was severely depressed and recumbent. Owners reported that the cat had showed anorexia and lethargy for three days. This animal lived in a rural area, having outdoor access and it was neither vaccinated nor treated against internal or external parasites. In addition, the cat did not receive any treatment before arriving at the clinic.

**Fig. 1:**
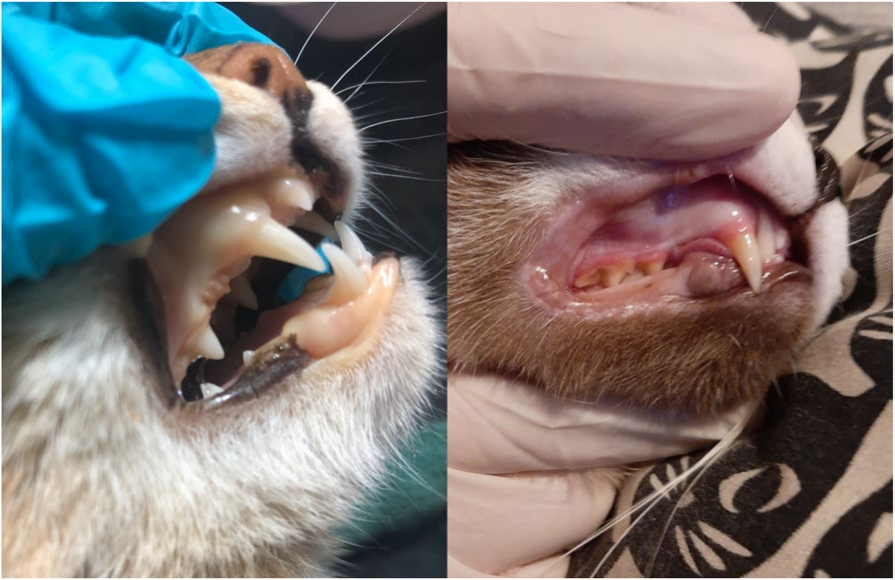
Close examination of the patient’s mouth showing pale and jaundiced mucous membranes (left) compared with a healthy cat (right)

The clinical examination revealed pale and jaundiced mucous membranes (Fig. 1) as well as an increase in both body temperature (40.4ºC) and respiratory and heart rates. The estimated degree of dehydration was 10%. Nothing unusual was detected on the thoracic radiography, and ultrasound examination revealed that the cat was pregnant; the gestation period was around 21 days.

Haematological parameters revealed haemolytic regenerative anaemia (hematocrit 12.8%, haemoglobin 4.3 g/dl and reticulocytosis), thrombocytopenia (26.000 platelets/µl) and leukocytosis (22.100 leukocytes/µl). A biochemical analysis determining glucose, creatinine, blood urea nitrogen (BUN), alanine transaminases (ALT), alkaline phosphatase (ALKP) as well as total protein, albumin and globulins was also performed; all parameters were within the normal range except for lower levels of creatinine and ALKP (Table 1).

**Table 1:** Haematological and biochemical results of the patient on the day it arrived to the clinic

	**Results**	**Reference Range**
**Hematocrit (%)**	**12.8**^a^	24.0–45.0%
**Haemoglobin (g/dL)**	**4.3**^a^	8.0–15.0
**Mean corpuscular haemoglobin concentration (g/dL)**	33.6	30.0–36.9
**Reticulocytes (%)**	> 4.0	
**Leucocytes (**10^3^/µL)	**22.10**^a^	5.00–18.90
**Granulocytes (**10^3^/µL)	6.2	2.50–12.50
**Granulocytes (%)**	28.1	
**Lymphocytes/Monocytes (**10^9^/L)	**15.9**^a^	1.5–7.8
**Lymphocytes/Monocytes (%)**	72	
**Platelets (**10^3^/µL)	**26**^a^	175–500
**Glucose (**mg/dL)	123	74–159
**Creatinine (**mg/dL)	**0.4**^a^	0.8–2.4
**Blood urea nitrogen (BUN) (**mg/dL)	25	16–36
**BUN/Creatinine**	60	
**Total Protein (**g/dL)	6.1	5.7–8.9
**Albumin (**g/dL)	2.2	2.2–4.0
**Globulin (**g/dL)	3.9	2.8–5.1
**Albumin/Globulin**	0.6	
**Alanine aminotansferase (ALT) (**U/L)	67	12–130
**Alkaline phosphatase (ALKP) (**U/L)	** < 10**^a^	14–111

^a^not into the normal range

Considering the clinical and haematological parameters, the animal was orally treated with doxycycline hyclate at a dose of 5 mg/kg (Vibracina 10 mg/ml, Hospira Invicta, Alcobendas, Spain) and prednisone at 0,5 mg/kg (Prednisona Cinfa 2,5 mg, Cinfa S.A., Huarte, Spain) every 12 h. In addition, intravenous fluid therapy was administrated (Ringer Lactate, Braun Veterinaria, Barcelona, Spain). The patient was then hospitalised. However, after a slight clinical improvement, the animal's general state of health worsened; hematocrit was 6%. The owners did not agree to perform a blood transfusion, and the cat died on 12th of March. Unfortunately, necropsy was not performed.

Although no ticks were found during the external examination of the animal, north-western Spain is an endemic area for canine babesiosis [19] and ticks are abundant in this region [20]. For these reasons together with the clinical, epidemiological and haematological features, the presence of haemoparasites was suspected. Before the death of the animal, blood was collected from cephalic vein and thin blood smears were made. Smears were dried, fixed, stained with a commercial kit (Quick Panoptic, QCA S.A., Amposta, Spain) and examined under microscope at 1,000 × magnification. No parasitic forms were detected in the smear. The blood sample was then sent to the laboratory of the INVESAGA group (University of Santiago de Compostela, Lugo) for molecular analysis. For molecular detection of piroplasms, DNA was first extracted from 200 μl of blood using a commercial kit (High Pure PCR Template Preparation Kit, Roche Diagnostics GmbH®, Mannheim, Germany) following the manufacturer’ instructions. Subsequently, two conventional PCR protocols targeting the 18S rRNA gene and the ITS1 of *Babesia* spp. and *Theileria* spp. were performed as previously reported [21–23]. DNA of *B. vulpes* obtained from a dog and nuclease free water were included as positive and negative controls, respectively. Amplification was only observed for the 18S rRNA gene. The obtained product was purified and sequenced in both senses on an ABI 3730xl® DNA analyzer (Applied Biosystems, Foster City, CA, USA) using a Big dye Terminator v3.1 cycle sequencing kit® (Applied Biosystems, Foster City, CA, USA) at the Sequencing and Fragment Analysis Unit of the Santiago de Compostela University (Spain). The sequence was aligned and edited using ChromasPro® (Technelysium, Brisbane, Australia), and scanned against the GenBank database using the Basic Local Alignment Search Tool (BLAST; http://blast.ncbi.nlm.nih.gov/Blast.cgi). This 18S rRNA sequence (558 bp) shared 100% of identity to that of *B. canis* (EU622793.1) obtained from a dog in Poland [24]. This sequence was deposited in GenBank under accession number OM314918.

For assessing the possible existence of co-infections with other vector-borne pathogens, the DNA sample was analysed using different commercial qPCR Kits (EXOone®, Exopol, Zaragoza, Spain) for the detection of *Anaplasma* spp., *Borrelia burgdorferi* s.l., *Ehrlichia* spp., *Rickettsia* spp., *Mycoplasma* spp., *Hepatozoon canis*, *Cytauxzoon* spp., *Bartonella* spp. and feline leukemia virus (FeLV) proviral DNA. However, DNA of those pathogens was not detected in the blood of the cat.

## Discussion and conclusions

The clinical signs, the epidemiological data and the molecular results suggest that this is the first report of a fatal case of feline babesiosis caused by *B. canis.* This *Babesia* species is, together with *B. vulpes*, the most commonly detected species in dogs from north-western Spain [19]. In addition, the animal had a high probability of being bitten by ticks since it had outdoor access in a rural area and it was never treated against internal or external parasites. Infection of cats with canine *Babesia* species have been previously molecularly confirmed in southern areas of Italy, Spain and Portugal where canine babesiosis is endemic [9–12, 25]. Thus, *B. canis* infection was detected in one and eleven cats from Spain and Portugal, respectively [9, 10], *B. vogeli* in 44 cats from Portugal [10, 11, 25] and *B. vulpes* in two cats from Portugal and Italy [9, 12]. In addition, a *Babesia* species showing a 95% of identity when compared to *B. canis* deposited sequences was detected in a feline babesiosis case in Poland [8]. However, most of these animals were asymptomatic; only one cat out of the twelve positive to *B. canis* (8.3%), ten out of the 44 (22.7%) cats positive to *B. vogeli* [10] and the cat analysed in Poland [8] showed clinical signs compatible with piroplasmosis. The clinical signs observed in these animals included pyrexia, anaemia, weakness and hematuria [1]. In Europe, clinical piroplasmosis has been also previously described in blood smear positive cats without molecular identification of the parasite; thus, large *Babesia* species were detected in cats with clinical babesiosis from France and Germany [6, 7] and small *Babesia* spp. were detected in a cat from France [17].

In *B. felis*-endemic areas of Africa it has been suggested that the predisposition for acquiring clinical babesiosis is lower in cats than in dogs [4]; in addition, it was reported that cats younger than three years are more predisposed to infection, suggesting an early exposure to infection as well as an age-related immunity [26], as it has been described for other piroplasm species [27–29]. Other factors increasing host susceptibility may be the existence of mixed infections with other pathogens and the presence of other immunosuppressive factors [9, 16]. In fact, six out of the eight cats (75%) positive to canine *Babesia* species detected in Portugal and Spain were also positive to feline leukemia virus (FeLV), feline immunodeficiency virus (FIV) or *Mycoplasma haemofelis* [9, 10]; it is worth noting that one of these animals presented a co-infection *B. canis/B. vogeli* [10]. Similarly, another study from Portugal reported that two (4.7%), three (7.0%) and eight (18.6%) out of 43 *Babesia*-positive animals were also positive to *Hepatozoon* spp., *Leishmania* spp. and *Borrelia burgdorferi* s.l., respectively [11]; in addition, two cats (4.7%) had a co-infection with Anaplasmataceae and *Hepatozoon* spp. [11]. In Israel, a symptomatic cat infected with *B. canis* subsp. *presentii* was co-infected with feline immunodeficiency virus and “*Candidatus* Mycoplasma haemominutum” [16]. In the current clinical case, the patient was negative to several vector-borne pathogens including *Anaplasma* spp., *Borrelia burgdorferi* s.l., *Ehrlichia* spp., *Rickettsia* spp., *Mycoplasma* spp., *Hepatozoon canis*, *Cytauxzoon* spp., *Bartonella* spp. and exogenous FeLV proviral DNA was not detected. However, the cat was young (six months old) and this, together with its pregnant state, may have increased its susceptibility for developing a clinical babesiosis [26].

Our results are consistent with those reporting that cats can be infected with canine *Babesia* species. Although there is evidence that most of these animals are asymptomatic carriers, some situations negatively affecting the immune system such as co-infections with other pathogens or the presence of immunosuppressive factors (i.e. gestation, immunosuppressive treatments…) may enhance the pathogenicity of piroplasms and therefore play an important role in the clinical outcome. For these reasons, *Babesia* spp. infection should be included in the differential diagnosis of animals with anaemia, thrombocytopenia, anorexia and lethargy, especially in young or immunocompromised animals from areas endemic for canine babesiosis.

## Data Availability

The authors declare that all data supporting the findings of this study are available within the article. Sequence data that support the findings of this study have been deposited in “GenBank” with the accession code OM314918.

## Abbreviations

BUN: Blood urea nitrogen
ALT: Alanine transaminase
ALKP: Alkaline phosphatase
FeLV: Feline leukemia virus
FIV: Feline immunodeficiency virus
